# Beyond the Surface: Deciphering the Role of Genetic Susceptibility in BIA-ALCL Pathogenesis

**DOI:** 10.3390/biomedicines14030600

**Published:** 2026-03-08

**Authors:** Young-Sool Hah, Seung-Jun Lee, Jeongyun Hwang, Hye Young Choi

**Affiliations:** 1 Biomedical Research Institute, Gyeongsang National University Hospital, Jinju 52727, Republic of Korea; yshah@gnu.ac.kr; 2 Department of Convergence Medical Sciences, Gyeongsang National University, Jinju 52725, Republic of Korea; 0789zxc@gnu.ac.kr (S.-J.L.); dkdl10252@gnu.ac.kr (J.H.); 3 Department of Radiology, Institute of Medical Science, Gyeongsang National University College of Medicine, Jinju 52727, Republic of Korea; 4 Department of Radiology, Gyeongsang National University Hospital, Jinju 52727, Republic of Korea

**Keywords:** BIA-ALCL, breast implants, textured implants, genetic susceptibility, gene-environment interaction, BRCA1/2, HLA, JAK/STAT, PD-L1, biofilm, foreign-body response, tumor microenvironment, precision prevention

## Abstract

**Background/Objectives:** Breast implant-associated anaplastic large cell lymphoma (BIA-ALCL) is the sentinel implant-associated malignancy, illustrating how long-lived biomaterials can reshape local tissue–immune ecology. Although textured (high-surface-area) implants show the strongest epidemiologic association, the rarity of disease despite widespread exposure suggests additional host modifiers. We synthesize evidence supporting a gene–environment (G × E) framework and critically appraise emerging host-susceptibility signals (including *BRCA1/BRCA2* and HLA associations). **Methods:** We conducted a narrative, evidence-based synthesis of peer-reviewed epidemiologic and registry studies, peri-implant niche biology (biofilm/foreign-body response and cytokine milieu), tumor genomic profiling, and current guidelines/regulatory communications, prioritizing primary studies for key claims. **Results:** Textured exposure dominates risk attribution, whereas absolute-risk estimates vary with denominators, exposure ascertainment, and follow-up duration. Mechanistic studies support a chronically inflamed capsule niche. Genomic analyses repeatedly converge on JAK/STAT pathway activation with frequent co-alterations in epigenetic regulators and recurrent copy-number changes, consistent with stepwise evolution under sustained selection. Immune-evasion features—including frequent PD-L1 expression and *CD274* (9p24.1) copy-number alterations—provide a plausible checkpoint route, while host-susceptibility signals remain preliminary and require multi-center, multi-ancestry replication. **Conclusions:** BIA-ALCL is a multistep, context-dependent lymphoma in which implant-mediated inflammation intersects with host susceptibility to enable somatic evolution and immune escape. Clinically, prevention currently relies on exposure mitigation, standardized risk communication, and symptom-driven evaluation; precision prevention will require integrative cohorts linking verified device exposure, immunogenetics, microenvironment profiling, and tumor multi-omics.

## 1. Introduction

Breast implants are among the most widely used medical devices in modern reconstructive and aesthetic surgery, with tens of millions of individuals estimated to be living with implants worldwide [1]. As surveillance of long-term implant outcomes has matured, a small but clinically consequential group of implant-associated malignancies has emerged, reframing implants not only as devices with mechanical complications but also as potential modifiers of local tissue–immune ecology [1,2]. Among these, breast implant-associated anaplastic large cell lymphoma (BIA-ALCL) has become the sentinel entity that has shaped clinical awareness, regulatory policy, and research priorities at the interface of biomaterials and lymphomagenesis [2,3,4,5].

BIA-ALCL was first reported in proximity to a breast implant in 1997, initiating a line of investigation that ultimately distinguished this clinicopathologic syndrome from systemic anaplastic large cell lymphoma [3]. Regulatory recognition accelerated after the U.S. FDA highlighted a possible association in 2011, catalyzing case consolidation, registry development, and standardized reporting [5,6]. In 2016, the World Health Organization incorporated BIA-ALCL into the classification of lymphoid neoplasms, formalizing it as a distinct disease entity and anchoring diagnostic expectations for pathology practice [4].

**Figure 1:**
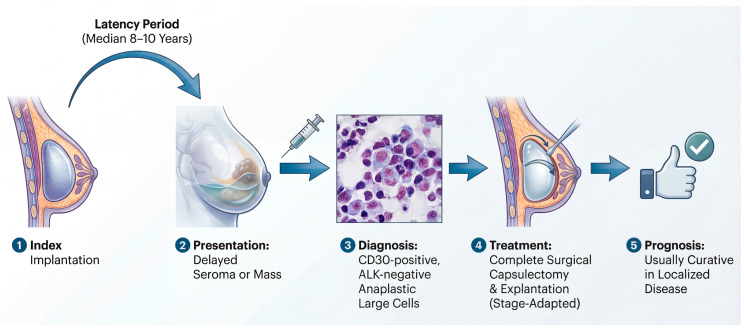
The typical clinical timeline of breast implant-associated anaplastic large cell lymphoma (BIA-ALCL): from implantation to management. The pathogenesis of BIA-ALCL is characterized by a prolonged latency period (median 8–10 years) following the implantation of a textured device. The disease most frequently presents as a delayed, unilateral periprosthetic fluid collection (seroma), or less commonly, as a capsular mass. Diagnosis requires aspiration of the fluid or biopsy of the mass, followed by cytologic and immunophenotypic analysis, which shows large, pleomorphic cells that are strongly CD30-positive and ALK-negative. For most patients with disease confined to the capsule (localized disease), complete en bloc surgical capsulectomy and implant explantation are the definitive treatments, which are usually curative and result in an excellent long-term prognosis.

Clinically, BIA-ALCL most often presents years after implantation as a delayed peri-implant seroma and/or a capsular mass, with tumor cells typically showing strong CD30 expression and lacking ALK expression [1,6,7]. This presentation is not merely descriptive; it tracks the biology and clinical behavior of the disease, which, in most patients, remains localized to the periprosthetic compartment and can be cured with complete surgical excision [7]. Contemporary consensus pathways therefore emphasize timely recognition, standardized cytologic and immunophenotypic assessment of effusions, and stage-adapted management rather than empiric escalation to systemic therapy [6,7]. Despite these remaining unknowns, the clinical definition of the disease has solidified. As illustrated in Figure 1, BIA-ALCL follows a characteristic clinical trajectory. It is a disease of long latency, typically emerging approximately a decade after the index implantation of a textured device. The hallmark presentation is a delayed, cold seroma, and diagnostic workup hinges on identifying CD30-positive, ALK-negative anaplastic lymphoid cells within the periprosthetic effusion or capsule. Crucially, this lymphoma exhibits a unique, stage-dependent biological behavior; most cases remain confined to the prosthetic capsule and are eminently curable with complete surgical excision alone.

Epidemiologically, the absolute risk of BIA-ALCL is low, but estimates vary substantially by geography, implant type, and ascertainment strategy, underscoring the limitations of passive surveillance and incomplete denominators [1,7,8]. The weight of population-based and registry evidence implicates textured-surface implants as the dominant exposure associated with BIA-ALCL, with risk appearing to increase with higher surface area texturing in multiple settings [9,10,11,12]. For example, a Dutch nationwide case–control analysis quantified a markedly increased relative risk of breast-ALCL among women with implants and derived non-trivial cumulative risks in an era when textured devices were commonly used [9]. Similarly, epidemiologic analyses from Australia/New Zealand, as well as the United States, report the highest risks among recipients of textured implants and highlight manufacturer- and surface-dependent heterogeneity that complicates patient counseling when exposure histories are incomplete [10,11].

Regulatory actions have mirrored—and in turn influenced—this evolving risk landscape [13,14]. In 2019, the FDA requested a voluntary recall of specific macrotextured devices (including Allergan BIOCELL) after accumulating evidence that suggested disproportionate representation among reported cases [13]. Subsequent FDA labeling initiatives—including boxed warnings and patient decision checklists—reflect a broader shift toward explicit risk communication for implants as non-lifetime devices requiring longitudinal surveillance [14]. More recently, FDA safety communications have also highlighted rare reports of other capsular malignancies (e.g., squamous cell carcinoma and additional lymphomas distinct from BIA-ALCL), reinforcing the need for careful pathologic evaluation of late seromas, masses, and atypical capsular findings [15,16].

Mechanistically, BIA-ALCL is increasingly viewed as a disease of chronic immune stimulation that occurs in a permissive microenvironment created by the periprosthetic capsule, where physical, chemical, and microbial cues may converge over time [6,7,17]. A leading model proposes that bacterial biofilms—potentially favored by textured surfaces—sustain local inflammation and T-cell proliferation, providing a substrate for malignant transformation in susceptible hosts [17,18]. Consistent with a multistep process, genomic studies reveal recurrent alterations that converge on JAK–STAT signaling and other oncogenic pathways, supporting a framework in which chronic stimulation is coupled to acquired driver events [19,20].

Despite rapid advances, fundamental uncertainties remain, including device-specific risks across markets, the contribution of host genetics and immune context, and the extent to which modifiable perioperative factors (e.g., contamination mitigation) can meaningfully reduce incidence [7,8,17,18]. In this Review, we synthesize current evidence on epidemiology and exposure patterns, pathobiology and genomic drivers, diagnostic and staging approaches, and management strategies, with a focus on translating heterogeneous data into practical, evidence-graded guidance for clinicians and researchers [6,7,8]. To construct this narrative synthesis, we performed a comprehensive literature search in PubMed, Scopus, and Web of Science from inception to December 2025. Search terms included ‘BIA-ALCL’, ‘pathogenesis’, ‘genetics’, and ‘epidemiology’. We prioritized primary studies (clinical trials, prospective cohorts, registry data) and peer-reviewed articles in English. Given the heterogeneity in epidemiological estimates across regions and implant types, we focused on highlighting established consensus and on critically evaluating conflicting data rather than pooling data for meta-analysis.

## 2. The Current Landscape: Limitations of the Environmental Model

### 2.1. The Texture–Biofilm Paradigm: Compelling, but Not Yet Sufficient

Textured implant surfaces have long been considered the key environmental variable in BIA-ALCL, largely because they can increase the interface area for tissue ingrowth, frictional wear, and microbial adherence [9,11]. In an influential multi-center analysis, Hu and colleagues detected bacterial biofilm in peri-implant capsules from BIA-ALCL patients and compared these to capsules removed for high-grade capsular contracture, revealing both a high bacterial burden and differences in microbiome composition between the groups [18]. This work is often interpreted as supporting a “biofilm-driven chronic antigen stimulation” model, in which persistent microbial and/or particulate cues maintain T-cell activation until a rare malignant escape occurs [18,21]. Importantly, however, the same study also underscores a central ambiguity: biofilm was demonstrable in both malignant and non-malignant capsule settings, so presence alone cannot discriminate between cause and background colonization [18].

A second line of evidence supporting an environmental axis predates frank lymphoma and instead tracks immunologic “priming” around contaminated implants [21]. Hu et al. previously reported that chronic biofilm infection in breast implants is associated with an increased T-cell lymphocytic infiltrate, providing a plausible mechanistic link between device colonization and sustained immune activation [21]. In parallel, experimental work has shown that implant surface characteristics influence biofilm formation in vitro and in vivo, consistent with the idea that topography can tune microbial adherence and persistence [22].

Yet, even taken together, these data remain associative rather than determinative for at least three reasons [18,22]. First, most capsule studies are cross-sectional at explant and therefore cannot establish whether specific microbial states precede (rather than follow) oncogenic transition [18]. Second, measurement methods are method-dependent (qPCR, sequencing, FISH, SEM), and low-biomass contamination or sampling heterogeneity can bias the apparent “signature” toward spurious separation [18]. Third, and most consequential for causality, the biofilm model does not, by itself, explain why a ubiquitous exposure (implant-associated colonization and chronic inflammation) yields a vanishingly rare malignancy [1,18].

### 2.2. Epidemiology: Strong Association Without Determinism

Across multiple national datasets, the risk of BIA-ALCL is disproportionately concentrated among patients exposed to textured devices, particularly those with higher surface areas [9,11]. In Australia and New Zealand, Loch-Wilkinson et al. reported that all identified cases had textured implant exposure, and that higher-surface-area textured devices were associated with a markedly increased risk compared with lower-surface-area textures [11]. To provide a clearer view of the evidence linking specific surface modifications to disease risk across different populations and study designs, key epidemiologic findings are summarized in Table 1. In the Netherlands, a population-based case–control study by de Boer et al. quantified a strong association between breast implants and breast ALCL, providing estimates of absolute risk using population denominator approaches [9]. Prospective institutional follow-up can yield higher cumulative incidence estimates than sales-denominator approaches. In a defined reconstruction cohort consistently followed over the long term, Cordeiro et al. observed BIA-ALCL cases after prolonged exposure to macro-textured devices [23].

However, the epidemiologic picture also exposes a core limitation of a purely environmental model: exposure is common, disease is rare, and the risk gradient—while real—does not behave like a single-variable switch [1,23]. Even in cohorts with macro-textured exposure predominance, only a small minority develop BIA-ALCL after a long latency, indicating that texture alone is insufficient for malignant transformation [6]. Meanwhile, passive surveillance systems capture important signals but are not incidence instruments: the FDA’s MDR summary (through 30 June 2024) explicitly notes that MDR data are subject to underreporting, duplicates, missing denominators, and therefore cannot be used to calculate true incidence or prevalence [13].

This “association without determinism” matters for how we interpret conflicting reports in the literature [9,13]. Studies can disagree not because one is “right” and the other “wrong,” but because they differ in denominator construction (sales data vs. registries vs. imaging-based prevalence), case ascertainment intensity, implant market composition, and duration of follow-up [9,23]. Accordingly, a high-level conclusion is that stable-macro-textured exposure carries a higher risk; however, the precise numerical risk estimate remains context-specific and sensitive to methodology [9,11,13,23].

### 2.3. The Missing Variable: Why Only a Minority Progress

**Figure 2:**
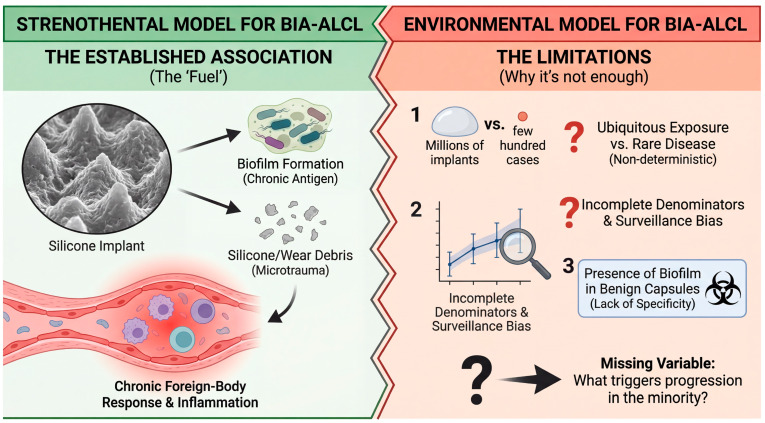
The “Texture–Biofilm” environmental paradigm: compelling association but incomplete causality. The left panel illustrates the currently established strong association (the “fuel”) between high-surface-area textured implants and chronic inflammation. Surface roughness promotes microbial adhesion (biofilm formation) and the generation of silicone particulate wear debris, both of which sustain a chronic foreign-body response and inflammatory infiltrate in the surrounding capsule. The right panel highlights critical limitations suggesting this environmental trigger alone is insufficient for malignant transformation. Despite tens of millions of women being exposed to textured implants worldwide, BIA-ALCL remains a rare disease, indicating a non-deterministic relationship. Furthermore, the interpretability of this association is constrained by surveillance bias, incomplete denominator data, and the observation that bacterial biofilms are also frequently detected on benign, non-tumor-associated capsules.

If surface topography and biofilm operate as environmental “fuel,” they still do not define the spark—the host—and tumor-intrinsic events that permit escape from regulated inflammation into clonal lymphoma [18,23]. Indeed, the biofilm-centric framework is already compatible with a multifactorial view, as Hu et al. explicitly argue for an interplay among host background, implant factors, and microbial drivers to explain divergent outcomes, such as contracture versus lymphoma [18]. From a population perspective, the same asymmetry is unavoidable: implants are present in an estimated tens of millions of people worldwide, while confirmed BIA-ALCL cases number in the low thousands, implying strong constraints beyond exposure alone [1]. These constraints are most plausibly encoded in variables that the environmental model typically treats as “noise,” including immune set points, local tissue microecology, duration-dependent inflammatory remodeling, and the probability of acquiring enabling somatic lesions over time [1,23]. Crucially, the long latency observed in many cohorts is consistent with a stepwise process rather than a single exposure–response event, aligning BIA-ALCL with other inflammation-associated lymphoid neoplasms that require both chronic stimulation and permissive genomic or epigenomic change [1,23]. However, the epidemiologic picture also exposes a core limitation of a purely environmental model. As summarized in Figure 2, while textured surfaces and their downstream consequences (biofilm, particulates, chronic inflammation) provide the necessary inflammatory “fuel,” they do not behave like a single-variable switch. Exposure is ubiquitous, yet the disease is rare. The presence of biofilm in benign capsules and the stark epidemiologic paradox—millions exposed versus thousands affected—strongly suggest that texture is an amplifier of risk rather than a deterministic cause, necessitating the search for additional variables that dictate progression in the minority.

In the next section, we therefore treat the environmental hypothesis as a necessary but incomplete first axis and ask what additional layers (genetic susceptibility, immunologic selection, and microenvironmental feedback) are required to convert chronic peri-implant inflammation into a CD30+ malignant clone [11,18,23].

## 3. Theoretical Framework: A Probabilistic “Two-Hit” Model for BIA-ALCL

**Figure 3:**
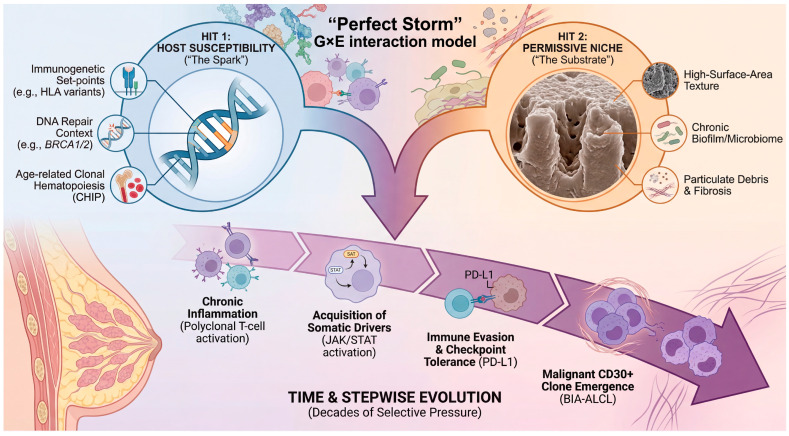
“Perfect Storm” theoretical framework: Gene–Environment (G × E) interaction in BIA-ALCL pathogenesis. This model proposes that BIA-ALCL emerges most efficiently when three distinct vectors converge. Hit 1: Host Susceptibility (“The Spark”) represents germline genetic backgrounds that raise the baseline risk, such as immunogenetic variations (e.g., HLA polymorphisms influencing antigen presentation), DNA repair defects (e.g., *BRCA1/2* carriers), or age-related pre-leukemic states (clonal hematopoiesis, CHIP). Hit 2: Permissive Niche (“The Substrate”) is the chronic inflammatory environment generated by the implant, driven by surface texture, biofilm, and particulate debris. Over decades of exposure (Time & Stepwise Evolution), this niche exerts sustained selective pressure on peri-implant lymphocytes. This promotes a stepwise somatic evolution, moving from chronic polyclonal inflammation to the acquisition of somatic drivers (e.g., JAK/STAT activation), immune evasion via checkpoint upregulation (PD-L1), and finally, the emergence of a malignant CD30+ clone. The arrows indicate the directional progression and interaction among host susceptibility, chronic inflammatory niche formation, stepwise somatic evolution, immune escape, and the eventual emergence of the malignant clone.

As summarized in Section 2, textured implant exposure is strongly associated with BIA-ALCL, yet the rarity of the disease indicates that exposure alone is insufficient. Building on this, we conceptualize BIA-ALCL within a two-hit/gene–environment (G × E) framework in which host susceptibility shapes baseline risk, while a chronic peri-implant inflammatory niche provides sustained selective pressure that enables stepwise somatic evolution and immune escape. This pattern is well captured by a “two-hit” logic in which a permissive host context and a sustained local selective pressure jointly enable malignant evolution [24]. Importantly, in BIA-ALCL, the “hits” need not map rigidly onto classic biallelic tumor suppressor inactivation; rather, they can be conceptualized as (i) a susceptibility axis that raises the baseline probability of transformation, (ii) an implant-centered inflammatory niche that promotes clonal selection and acquisition/expansion of oncogenic programs [24,25,26]. To resolve this paradox, we propose an integrated “two-hit” or “perfect storm” framework for BIA-ALCL pathogenesis (Figure 3). In this model, the implant is not a lone carcinogen but rather creates a permissive inflammatory niche—the environmental “substrate”—that sustains a chronic immune response over many years. Disease emergence, however, requires the intersection of this substrate with host susceptibility—the genetic “spark.” This G × E interaction sets the stage for a Darwinian evolutionary process within the capsule, in which sustained selective pressure allows rare T-cell clones to acquire the necessary somatic drivers and immune-escape mechanisms over decades, eventually leading to overt malignancy. Throughout this Review, the two-hit/G × E framework is presented as a working, hypothesis-generating model that organizes epidemiologic, immunobiologic, and genomic observations into testable predictions. While available evidence supports key components (e.g., textured exposure association; recurrent pathway-level selection such as JAK/STAT activation; immune-escape features), the framework does not yet constitute a validated, individual-level risk prediction tool, and causal inferences should be interpreted with appropriate caution.

### 3.1. First Hit: Host Susceptibility as a Genetic–Immunologic Set-Point

First, a “background” hit can be framed as host susceptibility, encompassing germline genetics, immune tone, and tissue responses that shape how the peri-implant capsule responds to chronic stimulation [25,26,27]. Historically, direct evidence that germline predisposition contributes to BIA-ALCL has been limited; however, recent cohort-based data now support the plausibility of an inherited risk in at least a subset of patients [28]. In a reconstruction cohort restricted to textured-implant exposure, carriers of pathogenic *BRCA1/2* variants showed a markedly increased hazard of implant-associated lymphoma compared with non-carriers, suggesting that DNA-repair context (or closely linked host factors) can modulate disease probability [28]. These findings should be interpreted cautiously, as they arise from a specific, clinically ascertained population and may reflect correlated exposures or surveillance; however, they nonetheless move “genetic predisposition” from conjecture to a testable, stratifiable variable [28].

Beyond BRCA, case-based observations further support a broader “susceptibility” concept that encompasses tumor-predisposition syndromes, although such evidence remains anecdotal and cannot define population risk [29]. Accordingly, the most defensible current model treats “first hit” as an immune and tissue-engineering phenotype, referring to the extent to which a given host forms a capsule, recruits and polarizes macrophages, and sustains T-cell-rich inflammation in response to an implanted biomaterial [25,26,27].

Mechanistically, multiple groups have converged on a BIA-ALCL immune signature consistent with an activated helper T-cell state, with cytokine profiling of cell lines and clinical specimens implicating a predominant Th17/Th1 program [30]. At the same time, histologic and cytokine findings also point to a concurrent allergic-type axis (including IL-13 biology), highlighting that baseline immune set-points may differ among patients and may shape the inflammatory “trajectory” of the capsule over years [30,31]. Consistent with this, IL-13 production by tumor cells has been reported in BIA-ALCL specimens, providing a plausible link between chronic inflammation, CD30 biology, and sustained survival signaling [31,32].

### 3.2. Second Hit: Implant-Centered Chronic Inflammation as a Long-Duration Selective Pressure

If host susceptibility sets the stage, the second hit is best conceptualized as the implant-centered niche—a long-lived, spatially confined environment that can sustain antigenic and innate immune stimulation [25,26,27]. Epidemiologic datasets consistently associate BIA-ALCL with textured devices (particularly those with higher surface areas), supporting the notion that microtopography and tissue integration can amplify the persistence of local immune activation [9,10,11,23]. At the tissue level, textured surfaces can drive the formation of thicker, more cellular capsules and more pronounced inflammatory responses in experimental systems, providing a biological substrate for prolonged immune cell trafficking and activation [33]. Within this niche, several non-exclusive stimuli have been proposed to sustain chronic signaling, including bacterial biofilm, particulate shedding, and ongoing foreign-body response dynamics [18,25,26,27]. Among these, bacterial biofilm has been directly detected in BIA-ALCL-associated specimens and contrasted with non-tumor capsules, supporting biofilm as a plausible contributor to chronic antigenic load (while not establishing causality) [18]. Crucially, regardless of the initiating stimulus, the “two-hit” framework predicts that the niche operates as a Darwinian filter, preferentially expanding rare T-cell clones that acquire survival and proliferation advantages under cytokine and checkpoint pressures [19,25,34,35].

Genomic studies provide strong support for this evolutionary view, repeatedly identifying oncogenic alterations centered on JAK/STAT signaling and cooperating lesions that affect transcriptional and epigenetic control [19,34,35]. Whole-exome sequencing first highlighted activating JAK1 and STAT3 mutations in BIA-ALCL, directly linking capsule-localized disease to the cytokine-responsive growth circuitry [34]. Subsequent series expanded this landscape to include recurrent STAT3 activation, copy-number changes, and additional candidate drivers (including *MYC*- and *TP53*-related abnormalities), consistent with convergent evolution toward sustained proliferative signaling and stress tolerance [19]. In a larger network-assembled cohort, alterations in epigenetic modifiers were frequent and often accompanied by lesions in the JAK/STAT pathway, suggesting that transcriptional reprogramming and cytokine signaling cooperate during the progression from in situ to invasive disease [35]. Notably, genetic subtyping studies support the notion that BIA-ALCL is typically negative for canonical ALCL rearrangements (e.g., *ALK*, *DUSP22*, *TP63*), reinforcing that the implant-associated entity is molecularly distinct and likely shaped by its unique niche [20].

Immune escape may represent an additional, niche-selected program, as frequent PD-L1 expression and recurrent *CD274* (9p24.1) copy number alterations have been reported, plausibly synergizing with constitutive pSTAT3 signaling [36]. This alignment—JAK/STAT activation plus checkpoint upregulation—offers a coherent mechanism by which a chronically inflamed capsule could both promote T-cell activation and ultimately select for clones capable of resisting immune-mediated deletion [35,36].

### 3.3. Latency: Time as an Enabling Variable for Stepwise Evolution

A defining feature of BIA-ALCL is its long latency, with many case series and reviews placing the typical presentation years after implantation, consistent with a slow evolutionary trajectory rather than an acute carcinogenic insult [7,9,11]. This latency is expected under a two-hit model: the first hit elevates baseline susceptibility, while the second hit supplies long-duration selective pressure during which rare oncogenic configurations can arise and expand to clinical detectability [24,25,35]. Consistent with stepwise progression, clinicopathologic frameworks describe in situ/effusion-limited disease and more invasive patterns, which can be interpreted as successive stages of niche-constrained evolution [7,37].

Clinical observations around implant exchange further support “time-and-niche” dynamics: in one analysis, patients with implant substitution had longer intervals from the first implantation to diagnosis, but a shorter interval from the most recent implantation to diagnosis, implying that exchange may reshape (rather than erase) the risk landscape [38]. Such patterns are compatible with the idea that a susceptible host may carry forward a propensity to re-establish an inflammatory capsule, while the selective process effectively “restarts” in a renewed niche [27,38].

Finally, placing BIA-ALCL within the broader canon of inflammation-linked lymphomagenesis helps calibrate expectations: chronic antigenic stimulation can generate lymphoid neoplasia only after prolonged exposure and intermediate steps, as exemplified by infection-associated and inflammation-associated lymphomas [39,40]. In BIA-ALCL, the implant capsule may be viewed as an engineered, long-lived “immune organ” in which sustained stimulation, somatic diversification, and immune escape can—rarely—align to produce malignant CD30+ T-cell outgrowth [26,30,35,36].

## 4. Genomic Drivers and Molecular Evolution of BIA-ALCL

### 4.1. A Constrained but Non-Random Genomic Landscape

Although an anatomically and etiologically distinctive niche defines BIA-ALCL—arising at the implant–capsule interface—its tumor genome is not “quiet” so much as constrained, with recurrent lesions converging on a limited set of signaling and regulatory axes [20,35,41]. Genetic subtyping analyses indicate that BIA-ALCL is typically negative for canonical ALCL rearrangements (e.g., *ALK*, *DUSP22*, *TP63*), supporting the view that the implant-associated entity is molecularly distinct and likely shaped by its unique niche [20]. Accordingly, BIA-ALCL appears to be a disease in which context (chronic local immune stimulation) and pathway selection may outweigh a single unifying structural driver [20,42].

### 4.2. Recurrent JAK–STAT Activation as a Central Organizing Principle

**Figure 4:**
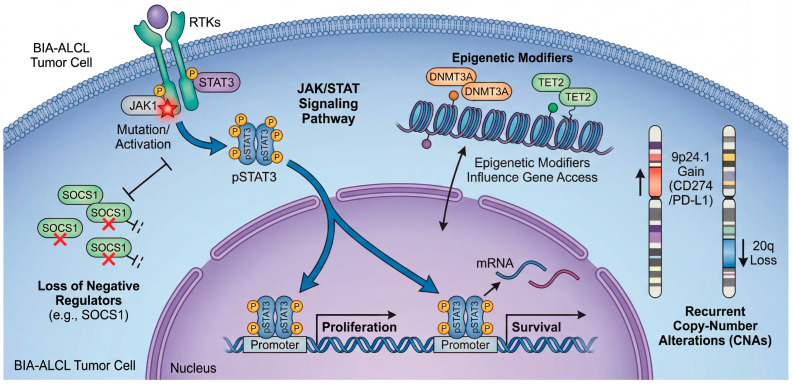
A constrained but non-random genomic landscape converging on constitutive STAT3 activity. Genomic profiling of BIA-ALCL reveals recurrent alterations that cooperatively drive sustained proliferation and survival signals, centering on the JAK/STAT pathway. The diagram illustrates an aberrant BIA-ALCL tumor cell. Constitutive activation of STAT3 (pSTAT3) is achieved through multiple converging mechanisms: activating somatic mutations in *JAK1* or *STAT3* itself; loss-of-function alterations in negative regulators such as *SOCS1*; and epigenetic modifications (involving regulators like DNMT3A or TET2) that influence gene accessibility. Furthermore, recurrent copy-number alterations (CNAs) reinforce this state, including gains at 9p24.1 (encoding CD274/PD-L1) and losses at 20q. These diverse genomic hits converge into a common transcriptional program that supports tumor cell survival in the inflammatory niche. The colors distinguish the major functional categories of alterations, including direct JAK/STAT pathway activation, loss of negative regulation, epigenetic dysregulation, and copy-number alterations, while the symbols indicate the type of genomic event represented in each component of the model.

Across independent sequencing efforts, JAK–STAT activation emerges as the most reproducible molecular theme in BIA-ALCL [19,34,35,43]. Early whole-exome sequencing (WES) first highlighted activating mutations in *JAK1* and *STAT3*, providing direct genetic evidence that constitutive STAT3 signaling can be hardwired into the tumor clone [34]. Subsequent comprehensive genomic profiling reinforced both the prevalence and diversity of genetic routes leading to this state: in one cohort analyzed with a broad haemato-oncology panel plus copy-number assessment, sequence variants consistent with JAK/STAT activation were highly prevalent, with activating *STAT3* mutations detected in the majority [19]. Beyond *STAT3* itself, lesions affecting negative regulators (for example, truncating alterations in *SOCS1*) or phosphatase control plausibly sustain pathway output, underscoring that BIA-ALCL selects for persistent STAT3 transcriptional competence rather than a single invariant mutation [19,44]. This convergence mirrors broader biology in ALK-negative ALCL, where STAT3 activation can be achieved through convergent mutations or kinase fusions. However, the apparent ubiquity of JAK/STAT lesions in BIA-ALCL suggests that the periprosthetic microenvironment may exert unusually strong selective pressure on this signaling pathway [19,45]. Across independent sequencing efforts, the most reproducible molecular theme is a potent convergence on STAT3 signaling. As depicted in the genomic landscape map in Figure 4, BIA-ALCL tumor cells employ a diverse array of genetic and epigenetic mechanisms to achieve the same end: constitutive STAT3 transcriptional competence. This includes direct activating mutations in *JAK1* and *STAT3* itself, disabling mutations in negative feedback regulators like *SOCS1*, and cooperating epigenetic dysregulation. This striking redundancy suggests an unusually strong microenvironmental selective pressure forcing the tumor clone to maintain this specific signaling hub.

### 4.3. Functional Reinforcement of STAT3 Programmes in Model Systems and Transcriptomes

Genetics is complemented by functional work in BIA–ALCL-derived model systems. Studies establishing and profiling BIA-ALCL cell lines have identified cytokine-dependent survival pathways and highlighted pathway-level vulnerabilities consistent with a STAT3-centered state [46,47]. At the transcriptome level, expression profiling distinguishes BIA-ALCL from other peripheral T-cell lymphomas while simultaneously revealing shared ALCL features, including signatures consistent with STAT3 activation and down-modulation of T-cell receptor signaling—an observation that fits a model in which chronic antigenic or innate inflammatory cues substitute for classical TCR-driven activation in sustaining malignant fitness [48]. New model development continues to expand experimental tractability; a recently described BIA-ALCL model (BIA-XR1) again supports JAK/STAT engagement (*STAT3* mutation) and raises the possibility that secondary pathways (e.g., RAS/MAPK) can occasionally be co-opted, although generalizability will require validation across larger primary cohorts [47].

### 4.4. Copy-Number Alterations: Chromosome-Scale Events with Entity-Level Specificity

If point mutations emphasize pathway convergence, copy-number alterations (CNAs) highlight entity-level specificity. In a 29-case series with genome-wide CNA profiling, CNAs were detected in 94% of BIA-ALCLs, and losses at 20q13.13 occurred in 66%, an alteration proposed as characteristic of BIA-ALCL relative to comparator ALCL cohorts [41]. These data suggest that BIA-ALCL is not merely a clinicopathological label applied to conventional ALCL arising near implants, but carries a reproducible chromosomal signature, and that genomic architecture may be shaped by chromosome-level dosage effects as much as by canonical focal drivers [20,41]. Additional CNA patterns reported across cohorts include recurrent focal deletions and amplifications with plausible functional consequences; for example, focal deletion involving *RPL5* and amplifications affecting loci such as *TNFRSF11A* (*RANK*), *PDGFRA*, and occasionally *MYC* have been described, pointing to heterogeneity in accessory lesions that may tune proliferation, inflammatory crosstalk, or stromal interaction [19]. Complementary sequencing approaches also suggest that lesions, such as 11q22.3 loss involving *ATM*, can appear in some tumors, implying that defects in the DNA-damage response may contribute to genomic instability or progression in a subset [49].

### 4.5. Epigenetic Regulators as Frequent Co-Targets

Beyond signaling and copy number, larger series emphasize that BIA-ALCL frequently harbors alterations affecting epigenetic modifiers, suggesting that chromatin deregulation is a second major axis of tumor biology [35]. In particular, integrated analyses framed the BI-ALCL landscape as characterized not only by JAK/STAT-activating mutations but also by loss-of-function alterations of epigenetic modifiers, supporting the selection of dual pathways [35]. Conceptually, this pairing is mechanistically attractive: chronic inflammatory niches provide persistent upstream cues (cytokines, innate immune ligands), while altered chromatin states stabilize downstream transcriptional circuits, locking cells into an activated, survival-competent phenotype even when extracellular signals fluctuate [35,48].

### 4.6. TP53: From Somatic Progression to Inherited Susceptibility

While most discussions focus on somatic drivers, several datasets raise a clinically consequential point: *TP53* alterations in BIA-ALCL may represent both somatic evolution and inherited susceptibility in a subset of rare patients [19,50]. In one genomic series with tumor–normal comparisons, germline *TP53* mutations were reported in a minority of cases, prompting discussion of the implications for risk estimation in predisposition settings (e.g., Li-Fraumeni syndrome) undergoing implant-based reconstruction [19]. Separately, targeted sequencing in a small BIA-ALCL series identified mutations not only in JAK/STAT pathway genes but also in *TP53* and *DNMT3A*, supporting the view that tumor-suppressor disruption and epigenetic-modifier lesions can emerge as additional somatic events in some cases [50]. Clinicopathological series describing distinct outcome groups in implant-associated ALCL underscore that a subset behaves aggressively (mass-forming, invasive, or disseminated), motivating the hypothesis that the accumulation of genomic complexity—including the disruption of tumor-suppressor pathways—may help explain the poor-outcome tails, even if definitive genotype–phenotype mapping remains incomplete [41,42].

### 4.7. Age-Related Clonal Processes and Interpretation Pitfalls (CHIP-like Lesions)

A recurring interpretive challenge in BIA-ALCL genomics is that several genes reported as altered (notably epigenetic modifiers such as DNMT3A) are also known to be canonical drivers of age-related clonal hematopoiesis (CHIP) [50,51]. Large-scale population sequencing studies have demonstrated that clonal hematopoiesis increases with age, often involving mutations in *DNMT3A*/*TET2*/*ASXL1*, and is associated with an elevated risk of subsequent hematologic malignancies and mortality. This provides a framework for understanding why CHIP-like variants may appear in tumor sequencing, particularly when matched normal tissue is unavailable [52,53]. Accordingly, when BIA-ALCL reports include CHIP-associated genes, the decisive question is not whether such variants are present, but whether they are tumor-restricted and clonally linked to malignant cells. Therefore, to avoid misinterpreting age-related clonal hematopoiesis (CHIP) as lymphoma-driving events, we strongly advocate for rigorous validation strategies, including paired tumor-normal sequencing, variant allele fraction contextualization, and multi-compartment sampling (e.g., blood vs. tumor vs. capsule) [19,51].

### 4.8. Emerging Synthesis and Testable Hypotheses

Taken together, current data support a model in which BIA-ALCL is shaped by a periprosthetic inflammatory ecosystem that repeatedly selects for sustained STAT3 output, while CNAs and epigenetic dysregulation provide additional layers of transcriptional stabilization and phenotypic diversification [19,35,41]. The most pressing next steps are therefore (i) larger multi-omics cohorts with matched normals to resolve CHIP confounding and quantify true driver prevalence, (ii) integrated microenvironment profiling to connect genotype with inflammatory and stromal states, (iii) functional dissection of recurrent copy-number events (e.g., 20q loss) to define how chromosomal dosage contributes to a uniquely implant-associated lymphoma identity [35,41,48]. A consolidated summary of the recurrent somatic and germline alterations defining the BIA-ALCL genomic landscape, along with their pathogenic roles and potential clinical implications, is presented in Table 2.

## 5. Immune Surveillance Escape and Host Immunity Factors

BIA-ALCL is best understood as a disease of contextual risk: exposure to a textured implant and its chronic peri-implant inflammatory niche appears necessary, yet it is clearly not sufficient, as only a small fraction of exposed individuals develop lymphoma. This gap between exposure prevalence and disease rarity strongly argues for host-level modifiers—including germline immunogenetic variation and inter-individual differences in inflammatory set points—that shape how persistent antigenic stimulation is sensed, amplified, and ultimately tolerated or extinguished [56,57].

### 5.1. Germline Susceptibility: HLA Variation as a Plausible Gatekeeper of Risk

Among the most compelling candidate host factors are HLA polymorphisms, which govern peptide presentation and can bias adaptive responses toward tolerance, effective clearance, or chronic inflammation. In a prospective, hypothesis-driven analysis of HLA alleles in BIA-ALCL patients compared with regional population controls, HLA-A*26 was substantially less frequent among cases than in the background population, suggesting that specific HLA configurations may modulate susceptibility (or, conversely, confer relative protection) [57].

Importantly, the interpretability of current HLA signals is constrained by the intrinsic limitations of rare-disease cohorts: small sample sizes, ancestry structure, and the possibility that HLA is acting as a proxy for linked immunoregulatory loci [57]. Nevertheless, the finding is conceptually aligned with broader lymphoma biology, in which antigen presentation and immune selection pressure can shape both the emergence and the immune visibility of malignant clones. In BIA-ALCL specifically, HLA variation offers a testable framework: distinct antigen-presentation landscapes could determine whether biofilm- and damage-associated stimuli around textured devices culminate in self-limited inflammation versus persistent T-cell activation with selective survival of aberrant CD30+ clones [56,57].

A near-term priority is therefore not simply “more genotyping,” but integrated immunogenetic epidemiology: multi-ethnic, well-phenotyped cohorts that align implant surface exposure, duration, clinical presentation (seroma-limited vs. invasive), and molecular subtype with germline HLA and other immune-regulatory variants [57]. Such datasets would enable the formal modeling of HLA-by-exposure interactions and facilitate clinically useful risk stratification only if effect sizes prove robust and reproducible.

### 5.2. The Peri-Implant Cytokine Niche: From Chronic Inflammation to Permissive Signaling

If germline variation influences the threshold for maladaptive immunity, the peri-implant environment provides the signal. A key insight from seroma-based profiling is that BIA-ALCL effusions carry a distinct cytokine signature, differentiating malignant from benign late seromas. In a multiplex cytokine study comparing BIA-ALCL-associated effusions with multiple categories of reactive seromas, elevated IL-10, IL-13, Eotaxin, and a discriminative IL-10/IL-6 ratio emerged as characteristic features of BIA-ALCL fluid [56]. These findings have spurred interest in developing non-invasive seroma screening panels. Key candidate biomarkers that may help distinguish malignant effusions from benign reactive seromas based on the underlying inflammatory microenvironment are detailed in Table 3.

These data matter both mechanistically and diagnostically. IL-10 is a canonical immunoregulatory cytokine that can dampen antigen-presenting function and effector T-cell activity, potentially creating a niche in which abnormal T-cell populations can persist [56]. IL-13 and Eotaxin point to a type 2/allergic-inflammatory axis, consistent with histologic observations of eosinophil-rich backgrounds in a subset of cases and raising the possibility that, in at least some patients, chronic peri-implant inflammation is not purely Th1/Th17-driven but includes a Th2-skewed component that can remodel tissue and immunity over time [31,56].

Notably, independent groups have continued to explore fluid-phase biomarkers for earlier and less invasive detection. A “real-world” clinical-translation effort reported that selected cytokines (including IL-9, IL-10, IL-13) can help discriminate malignant from benign seromas in peri-implant aspirates—an approach that is attractive because it leverages the disease’s most common presentation (delayed seroma) and can be implemented upstream of overt capsular invasion [58].

### 5.3. Tumor–Immune Co-Evolution: IL-13 Programs, Treg-like Features, and Attenuation of T-Cell Surveillance

Beyond soluble mediators, the cellular programs of BIA-ALCL suggest a dynamic interplay between inflammation-driven growth cues and adaptive immune pressure. Tumor-cell production of IL-13 has been demonstrated in BIA-ALCL cell lines and clinical specimens, linking the disease to pathways classically associated with allergic inflammation and tissue remodeling [31].

Mechanistic work further indicates that CD30 signaling can regulate components of an IL-13–STAT6 signaling axis in BIA-ALCL models, providing a plausible molecular bridge between the defining immunophenotype (CD30 positivity) and the cytokine circuitry that shapes the peri-implant niche [59]. While the translational implications remain early, these data motivate a hypothesis in which IL-13-dependent programs contribute to a permissive microenvironment—supporting survival, modulating stromal responses, and potentially biasing local immunity away from effective tumor clearance [31,59].

At the transcriptome level, BIA-ALCL appears molecularly distinct among peripheral T-cell lymphomas, with analyses reporting an activated CD4 memory-like phenotype and signatures consistent with immune modulation. Gene set enrichment has highlighted features such as downregulation of T-cell receptor signaling and STAT3 activation, as well as expression patterns compatible with CD25 and FOXP3 in many cases—observations that raise the possibility of Treg-like or immune-regulatory states that could blunt local antitumor immunity [48].

### 5.4. Immune Escape: PD-L1 as a Convergent Node and Emerging Microenvironment Atlases

**Figure 5:**
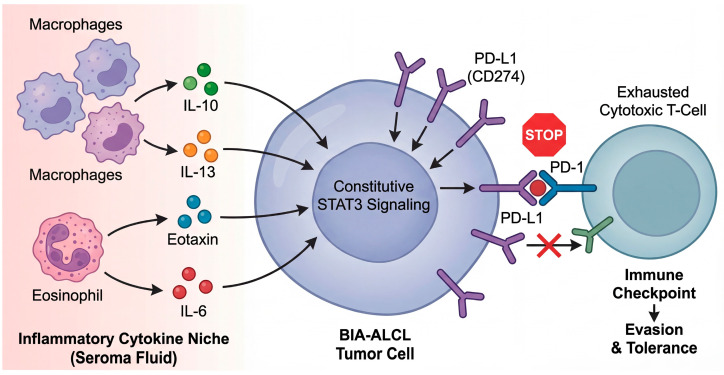
From chronic inflammation to immune escape: the cytokine-checkpoint axis in the periprosthetic niche. The peri-implant seroma fluid creates a unique cytokine milieu that shapes tumor evolution. The inflammatory niche, populated by macrophages and eosinophils, is enriched in cytokines such as IL-6, IL-10, and IL-13. These cytokines, particularly IL-13 and IL-6, act on the BIA-ALCL tumor cell to reinforce constitutive STAT3 signaling. In turn, STAT3 transcriptional activity drives the upregulation of the immune checkpoint ligand PD-L1 (CD274) on the tumor cell surface. The binding of tumor PD-L1 to PD-1 receptors on infiltrating cytotoxic T-cells provides a potent inhibitory signal, leading to T-cell exhaustion and tolerance, thereby allowing the malignant clone to escape immune surveillance within the capsular microenvironment.

The most direct molecular foothold for immune evasion in BIA-ALCL is the PD-1/PD-L1 axis. A detailed pathologic and genomic analysis reported frequent PD-L1 expression and recurrent *CD274* (PD-L1) copy number alterations at 9p24.1, suggesting that these lesions may share a common mechanism for PD-L1 upregulation, potentially acting in synergy with constitutive pSTAT3 signaling [36]. This is notable because it provides a genetically anchored route by which tumor cells may convert chronic inflammation into checkpoint-mediated immune suppression, thereby stabilizing malignant growth within an anatomically confined site [36]. Ultimately, the tumor must evade host immunity to survive. Emerging data indicate a dynamic coevolution between the tumor and its microenvironment, forming a critical cytokine-checkpoint axis (Figure 5). The seroma fluid is not merely passive, but is enriched with cytokines such as IL-10 and IL-13, which not only support tumor cell survival but also drive the expression of immune checkpoint molecules. The resultant upregulation of PD-L1 on tumor cells, acting on PD-1-positive infiltrating lymphocytes, establishes a “tolerogenic shield” that effectively converts the chronic inflammatory niche into a site of immune privilege.

Finally, the field is shifting from “marker-based” snapshots to systems-level microenvironment maps. Recent integrative analyses of the BIA-ALCL tumor immune microenvironment (including seroma-associated and invasive disease contexts) are being reported in hematology venues, offering early evidence that immune composition and functional states may differ across clinical stages [60]. Although much of this work is still emerging (often first appearing as conference abstracts), it is likely to refine how we think about immune surveillance in BIA-ALCL—shifting from a static notion of “chronic inflammation” to a model of co-evolution, in which immune editing and immune escape pathways become progressively more prominent as disease transitions from effusion-limited to invasive phenotypes [48,60].

In sum, the most coherent host-immunity model integrates: (i) a textured implant–conditioned inflammatory niche, (ii) germline immunogenetic context (with HLA as an early signal), (iii) tumor-intrinsic programs that reshape immunity through cytokine circuits (IL-13) and checkpoint activation (PD-L1). Translationally, these layers suggest two actionable directions: risk refinement (immunogenetics plus exposure metrics) and earlier detection (seroma biomarkers), while also providing a rational basis to explore immunomodulatory strategies in the minority of patients with invasive or refractory disease.

## 6. The Synthesis: Gene–Environment (G × E) Interaction

Breast implant-associated anaplastic large cell lymphoma (BIA-ALCL) is best understood as a device–tissue ecosystem failure in which persistent environmental stimulation intersects with host susceptibility, allowing for the stepwise somatic evolution of a malignant T-cell clone [9,10,11,12]. Across population-based and registry datasets, the strongest and most reproducible epidemiologic signal is the association with textured (particularly high-surface-area) devices, which likely function not as a singular carcinogen but as a long-lived scaffold that amplifies immune stimulation and tissue remodeling over years [9,10,11,12]. Genomic studies further indicate that BIA-ALCL is enriched for oncogenic lesions in cytokine signaling (notably JAK/STAT) and cooperating alterations in tumor suppressor/epigenetic regulators, consistent with selection within a chronically inflamed niche rather than a one-hit transformation event [18,19,34,35,50,61]. The long latency observed in large registries (often around a decade) is therefore biologically coherent: it provides the temporal window required for recurrent activation, clonal expansion, and selection of advantageous somatic variants under ongoing microenvironmental pressure [12]. In this framework, “G × E ” is not a slogan but a mechanistic proposition: implant surface properties and their downstream consequences (biofilm, particulate shedding, fibrosis, and cytokine tone) constitute the E, while immunogenetic variation, DNA repair capacity, and age-related clonal architecture constitute the G that tunes risk and trajectory [28,51,57,62,63,64].

### 6.1. From Friction to Mutation: Mechanotransduction and “Tribological Inflammation”

A distinctive feature of the peri-implant capsule is that it is not biologically static; it is a mechanically active, fibrotic structure that experiences cyclic loading, micro-motion, and—particularly for textured surfaces—high-friction interface dynamics, which can promote microtrauma and particle generation [63,64,65,66,67,68]. Recent tribology-focused experimental work supports the plausibility of this sequence by demonstrating that textured implant surfaces can generate wear debris under physiologically relevant motion regimes and that the resulting particulate exposure can elicit a pro-inflammatory macrophage response in vitro [65]. Independent pathology-oriented studies of human capsules further report detectable silicone particles in peri-implant tissues (with enrichment patterns that can differ by surface type), reinforcing the concept that the capsule may be repeatedly exposed to material-derived particulates over time [66,67,68]. Although the downstream consequences of silicone particulates in this specific disease context remain incompletely defined, the broader biomaterials literature demonstrates that particulates can function as danger-associated cues that amplify innate immune activation, including IL–1β–centered programs when appropriate priming signals are present [69,70,71].

Mechanotransduction provides an additional—and testable—axis linking “texture” to “tone” of inflammation [63,72,73]. In vivo and ex vivo data indicate that surface topography can shape the foreign-body response to silicone implants, including the architecture of fibrosis and the composition/activation state of immune infiltrates across model systems and human samples [63]. Consistent with this, analyses of periprosthetic fluid and capsule-associated immune signatures suggest that macrotextured surfaces can drive a chronic-like inflammatory state characterized by specific immune and molecular patterns, implying that surface design can bias the local cytokine milieu [64]. At the cellular level, macrophages are known to integrate mechanical inputs via transcriptional regulators such as YAP, and YAP activity can tune the magnitude of inflammatory responses to canonical stimuli—providing a plausible mechanistic bridge between a stiff/fibrotic capsule environment and heightened innate immune output [73].

How might these physical and inflammatory cues connect to the somatic genetic landscape of BIA-ALCL? Chronic cytokine exposure (for example, IL-6 family and related pathways) can converge on STAT3 signaling, and BIA-ALCL frequently exhibits constitutive JAK/STAT activation through recurrent somatic lesions, suggesting that a cytokine-rich niche could create strong selective pressure for clones that “lock in” growth/survival signaling [54,74]. In parallel, recurrent tissue injury and innate immune activation can increase oxidative and replication stress in proliferating lymphocytes, conditions known to elevate mutational opportunity even in the absence of exogenous mutagens [69]. A particularly intriguing mechanistic possibility—still hypothesis-generating in BIA-ALCL specifically—is that mechanical confinement and deformation within a dense fibrotic capsule could contribute directly to DNA damage in cycling cells, because physical squeezing can induce nuclear envelope rupture and associated DNA damage during confined migration, and nuclear deformation alone can drive replication-stress-linked DNA damage even without envelope rupture [75,76,77]. If peri-implant T cells undergo repeated activation, proliferation, and tissue trafficking within such a mechanically constrained niche, then the concept of “friction-to-mutation” becomes experimentally addressable. One would predict increased markers of DNA damage/replication stress in capsule-resident lymphocytes, as well as enhanced selection for lesions that attenuate checkpoint/apoptosis or potentiate cytokine signaling [75,76,77].

### 6.2. The “Perfect Storm” Scenario: Substrate, Spark, and Time

We propose that BIA-ALCL emerges most efficiently when three vectors converge: (i) a permissive substrate, (ii) a susceptible host (“spark”), (iii) sufficient time for evolution [9,10,11,12,28,51,57,62,63,64]. First, the substrate is created by a high-surface-area implant–capsule interface that can promote bacterial adherence and the formation of spatially complex biofilms. Biofilm-associated bacterial signatures (including *Ralstonia* spp.) have been reported in association with BIA-ALCL specimens in foundational studies that seeded this hypothesis [62]. In parallel, topography-dependent foreign-body responses can sculpt a fibrotic and immunologically active capsule, providing the “reactor vessel” in which chronic inflammation and local cytokine production persist [63,64]. Second, the spark is the host: immunogenetic variation may alter antigen presentation or immune set-points, and HLA expression/allelic distribution differences have been reported in BIA-ALCL cohorts, supporting the concept that host immunogenetics can modulate susceptibility [57]. More recently, a case–control analysis in women with breast cancer and textured implants reported a markedly increased risk of BIA-ALCL among *BRCA1/2* mutation carriers (including a large effect size), introducing DNA repair-linked host biology as a potential risk modifier that warrants replication and mechanistic exploration [28]. Host “spark” may also be conceptualized through the lens of age-related clonal hematopoiesis (CHIP), a prevalent state in which somatic clones expand with age and can increase the probability that a permissive inflammatory microenvironment will encounter pre-existing oncogenic or epigenetic lesions in hematopoietic lineages [51]. Third, time is essential: registry data demonstrate long exposure intervals before diagnosis and emphasize that the disease typically presents after years of implantation, consistent with a multistep evolutionary process under chronic selective pressure [12].

A key advantage of the perfect-storm model is that it yields clear prevention logic: removing any one vector should lower risk [9,10,11,12]. Indeed, the epidemiologic association with textured devices implies that reducing exposure to high-surface-area textures will attenuate the environmental substrate at the population level [9,10,11,12]. Similarly, interventions that reduce bacterial load/biofilm formation at implantation are mechanistically aligned with the substrate axis, even if the field still lacks definitive causal proof connecting any specific organismal community to malignant transformation [62]. Finally, recognizing host susceptibility factors—immunogenetic signals, DNA repair defects, and age-related clonal architectures—creates a path toward precision prevention, in which implant choice and surveillance intensity can be rationally aligned with individualized risk rather than uniformly distributed [28,51,57].

## 7. Clinical Implications: Towards Precision Prevention

The central clinical consequence of reframing BIA-ALCL as a gene–environment (G × E) disease is that “prevention” becomes more than device selection alone; it becomes a comprehensive program that integrates exposure reduction, structured surveillance, and risk communication proportional to the level of uncertainty [7,78]. At present, the only risk factor supported by convergent epidemiology and regulatory action is implant surface exposure, particularly macrotextured/high-surface-area devices, which have been selectively withdrawn or restricted in multiple jurisdictions [79,80,81,82]. By contrast, host-genetic modifiers remain plausible but not yet clinically actionable, as professional societies explicitly note that there is currently no validated test to identify who is at risk of developing BIA-ALCL [83]. Therefore, a precision-prevention strategy must be honest about evidence tiers: it should implement what is already supported (texture-focused exposure mitigation and standardized risk disclosure) while building the prospective datasets needed to validate genetic risk signals [14,84,85,86].

**Figure 6:**
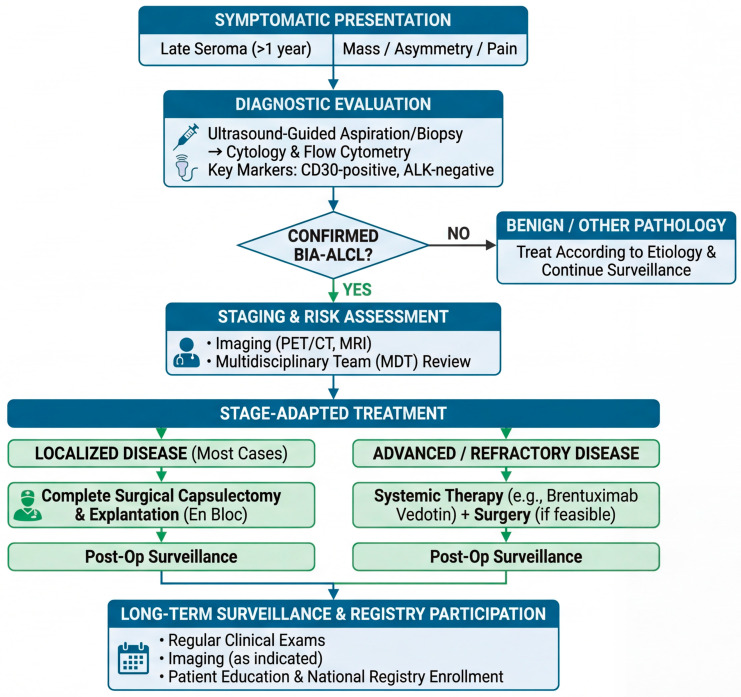
Proposed clinical management workflow for BIA-ALCL: from presentation to surveillance. This algorithm outlines a standardized approach to the diagnosis and management of BIA-ALCL based on current guidelines. The pathway begins with the recognition of key symptomatic presentations, primarily late seroma. Diagnostic evaluation relies on ultrasound-guided sampling for cytology and flow cytometry, with CD30 positivity being the cornerstone diagnostic marker. Confirmed cases require staging with cross-sectional imaging (PET/CT or MRI) and discussion in a multidisciplinary team (MDT). Treatment is strictly stage-adapted: complete en bloc capsulectomy is the standard of care for most patients with localized disease. Systemic therapies (e.g., brentuximab vedotin) are reserved for advanced or refractory cases. Long-term follow-up and contribution to patient registries are essential components of post-treatment care.

A second implication is that prevention and early detection are inseparable, because most cases are curable when recognized early and managed with guideline-concordant surgery and staging [1,11]. Accordingly, “precision” should be interpreted not only as molecular prediction but also as precision communication—ensuring that patients can recognize symptoms (late seroma, mass, asymmetry) and that care teams follow a reproducible diagnostic pathway [7,78]. A second critical implication of this new understanding is that early detection remains our most effective tool, as the disease is highly curable in its niche-confined state. We therefore propose a standardized clinical workflow to guide management (Figure 6). This pathway emphasizes the prompt evaluation of late seromas with targeted cytology (CD30 staining), rigorous staging for confirmed cases, and adherence to stage-adapted surgical protocols. Ensuring that care teams follow such a reproducible diagnostic and therapeutic pathway is fundamental to optimizing patient outcomes.

### 7.1. Proposal for a Pre-Operative Risk Stratification Workflow

Any preoperative risk stratification for BIA-ALCL must begin with a caveat: routine germline screening for BIA-ALCL susceptibility is not evidence-based today and should not be presented as a standard of care [83]. Nevertheless, the emerging literature supports a pragmatic intermediate goal—identifying patient groups in whom texture avoidance and follow-up intensity may be particularly important—while simultaneously enrolling these patients into registries and biobanks that can validate (or refute) candidate genetic modifiers [84,85,86]. It is important to emphasize that the risk stratification proposed below (and in Table 4) represents a provisional, precautionary framework based on emerging signals [28] and has not yet been formally adopted by major clinical society guidelines.

Tier 0 (universal): standardized exposure documentation and risk disclosure.

Before implant selection, clinicians should document the intended device characteristics (surface type, manufacturer, model) and provide patients with standardized risk information consistent with current regulatory expectations, including the FDA’s strengthened risk-communication actions (boxed warning/decision checklist requirements) [78,87]. This tier is not genetic, but it is foundational: without accurate exposure capture, downstream epidemiology and any future G × E modeling will remain underpowered and biased [84,85,86].

Tier 1 (clinical context): identify conditions where surgical alternatives may be preferred.

For all candidates, history should capture (i) reconstructive indication versus cosmetic augmentation, (ii) prior implant history and replacement cycles, (iii) immunologic or oncologic contexts that may influence counseling (e.g., prior lymphoma history or current immunosuppression), while acknowledging that direct BIA-ALCL-specific evidence for most host factors is limited [7,13,88]. Where feasible, this tier should also route patients toward prospective surveillance infrastructure (e.g., registry participation) rather than relying on retrospective recall after complications arise [84,85].

Tier 2 (genetic information when already clinically indicated): incorporate known cancer predisposition results into device choice.

A key principle is to use genetic results that are already being obtained for other clinical reasons, rather than ordering new “BIA-ALCL panels” without validation [28]. In breast cancer reconstruction settings, germline testing for *BRCA1/2* is frequently performed for oncologic decision-making. A recent cohort/case–control analysis reported a substantially increased risk of BIA-ALCL among *BRCA1/2* carriers with textured-implant reconstruction, raising a plausible DNA repair-linked susceptibility signal that warrants replication [28]. If such an association is confirmed, the immediate preventive translation would be straightforward: *BRCA1/2* carriers could be preferentially counseled toward smooth implants or autologous reconstruction when acceptable, with heightened symptom vigilance when textured implants are unavoidable [28,89]. More broadly, rare case reports (e.g., BIA-ALCL in Li-Fraumeni syndrome) support the conceptual possibility that canonical cancer predisposition states can intersect with implant exposures, but such reports should be treated as hypothesis-generating rather than decisive [29]. To further calibrate these emerging host-susceptibility signals, it is important to clarify the current evidentiary landscape. To date, we have not identified a published, adequately powered genome-wide association study (GWAS) specifically interrogating germline susceptibility to BIA-ALCL. The available data, therefore, consist primarily of cohort-specific association signals (e.g., HLA allele frequency differences) and clinical–genetic associations observed within defined reconstruction settings (e.g., *BRCA1/BRCA2*), which should be regarded as hypothesis-generating and in need of independent replication. A future GWAS/consortium effort would require multi-center case aggregation, rigorous verification of implant exposure histories (including surface type and duration), ancestry-matched controls, and harmonized phenotyping (effusion-limited vs. invasive disease) to minimize bias and enable interpretable risk modeling.

Tier 3 (research-only immunogenetics): HLA and immune-setpoint mapping.

HLA-based susceptibility signals have been reported (e.g., differential frequency of HLA-A*26 in a case series versus population controls), consistent with a model in which antigen presentation influences chronic immune activation at the implant–capsule interface [57]. However, given small sample sizes and population-structure concerns, HLA typing should be positioned as a research tool to enable prospective G × E modeling, rather than as a clinical screening test [57,83]. The practical deliverable for the field is therefore not an immediate clinical HLA test, but multi-center cohorts linking (i) exact device exposure, (ii) clinical phenotype (seroma-limited vs. invasive), (iii) tumor genomics with (iv) host immunogenetics [57,84,85,86]. To calibrate the strength of evidence across proposed host susceptibility signals, we stratify the supporting data into three tiers. Tier A (epidemiologic association) reflects convergent population-level evidence (e.g., the association between BIA-ALCL and textured, high-surface-area implants). Tier B (cohort-specific signal) includes associations observed within defined clinical cohorts (e.g., BRCA1/2-associated risk signals in reconstruction settings; HLA allele frequency differences) and remains provisional pending independent replication. Tier C (mechanistic plausibility) encompasses biological models supported by experimental or molecular data (e.g., chronic peri-implant inflammation, cytokine niche biology, and recurrent pathway-level selection, such as JAK/STAT activation), which provide coherence but do not, in themselves, establish causality or clinical predictability. It is essential to acknowledge that host susceptibility data are currently limited by small sample sizes inherent to rare diseases, potential ascertainment and surveillance bias in clinically followed reconstruction cohorts, incomplete or uncertain denominators and exposure histories, and lack of prospective validation. Accordingly, BRCA/HLA-related signals should be interpreted as hypothesis-generating and should not be used for routine clinical screening or risk stratification outside research settings until validated in multi-center, multi-ancestry prospective cohorts.

### 7.2. Patient-Tailored Implant Selection Guide: Minimizing Exposure While Preserving Reconstructive Goals

In the current evidence landscape, the most defensible preventive action is to minimize high-risk textured exposure, especially macrotextured/high-surface-area surfaces, because these have driven multiple market actions and public health restrictions [79,80,81,82]. The FDA’s 2019 recall notice for Allergan BIOCELL explicitly instructs that these products should no longer be implanted, reflecting regulator-level risk stratification by device category [79]. Similarly, France’s regulator (ANSM) issued a ban on macrotextured and polyurethane-coated implants in 2019, illustrating how prevention policy can be implemented at a national scale when risk–benefit is judged unfavorable [80]. Parallel actions and advisories from Australia’s TGA and Health Canada further underscore that the risk of implant surface is sufficiently consistent to inform regulatory restrictions across jurisdictions [81,82]. Given the evolving and sometimes divergent regulatory stances across different jurisdictions, clinician awareness of the current global landscape is essential for informed consent. A summary of key international regulatory actions regarding textured implants is provided in Table 5.

A practical selection matrix can be framed by two axes: (A) necessity of texture for the surgical objective, (B) host context.

When texture is not essential (most cosmetic augmentations and many reconstructive scenarios), smooth devices provide a prevention-aligned default that reduces exposure to the best-established risk factor [83,88]. When texture is considered for specific reconstructive goals (e.g., positional stability in certain anatomical designs), decision-making should explicitly document the incremental benefit of texture and discuss the known association between textured surfaces and BIA-ALCL [7,88]. For patients with a potentially higher-risk host context—most credibly those with known cancer predisposition results (e.g., BRCA1/2 carriers, pending replication)—the risk–benefit threshold for choosing textured devices should be higher, and alternatives (smooth implants, autologous options) should be discussed more strongly [28,78].

Counseling for patients with existing textured implants should prioritize individualized risk assessment rather than routine prophylactic explantation.

Multiple regulator- and society-linked statements emphasize that prophylactic removal of textured implants is not recommended for asymptomatic patients, because the disease is uncommon and surgical risks may outweigh uncertain benefit [90,91,92,93]. This guidance also implies a prevention opportunity: improving symptom literacy and follow-up pathways may reduce morbidity more reliably than pre-emptive surgery in the general asymptomatic population [7,90,91,92,93]. Where implant exchange or revision surgery is being performed for other indications, the procedure can be used as a structured “prevention touchpoint” to (i) update device documentation, (ii) renew informed consent under contemporary standards, (iii) re-evaluate whether any textured exposure remains necessary [78,87].

### 7.3. The Future of Informed Consent: From Complication Checklists to Biologic Risk Literacy

Informed consent is rapidly becoming the primary venue in which “precision prevention” is operationalized, because regulatory bodies now require more structured risk communication for breast implants [78]. The FDA’s 27 October 2021, actions strengthened risk communication through labeling expectations, including a boxed warning and a patient decision checklist, signaling a policy-level shift toward standardized, patient-facing disclosure [78,87]. As a consequence, consent should explicitly address not only surgical complications and rupture surveillance but also implant-associated malignancy risks—particularly BIA-ALCL—and the symptom patterns that should trigger evaluation (late seroma, mass, asymmetry, lymphadenopathy) [7,88].

Consent language should also reflect the expanding safety landscape beyond BIA-ALCL alone [15]. The FDA has issued safety communications regarding reports of squamous cell carcinoma and various lymphomas arising in the capsule around breast implants, emphasizing the importance of multidisciplinary evaluation and reporting, while noting that recommendations for BIA-ALCL management remain unchanged [15]. Even if these events remain very rare, their existence reinforces a broader counseling principle: implants are not inert devices but long-term interfaces that can shape tissue biology, and surveillance should be symptom-driven and continuous [15,78].

Finally, informed consent should be linked to data infrastructure, because prevention at the population scale depends on denominator-aware surveillance [84,85,86]. Participation in registries such as NBIR and disease-specific efforts such as PROFILE provides a mechanism to capture device characteristics, outcomes, and rare-event signals that cannot be reliably estimated from passive adverse-event reporting alone [84,85,86]. In parallel, consent processes should encourage patients to retain device information (manufacturer/model) and to seek evaluation promptly for late-onset changes, enabling earlier diagnosis and guideline-concordant care [7,78]. Integrating the concepts of exposure mitigation and emerging host susceptibility factors, Table 4 proposes a pragmatic, risk-stratified matrix to guide implant selection and surveillance strategies in the era of precision prevention. Consistent with the exploratory framework summarized in Table 4, future work should focus on moving from conceptual stratification to prospective validation in well-phenotyped, multicenter cohorts. Such studies should link verified implant surface–exposure histories and reconstruction context with host germline susceptibility signals and matched tissue/capsule/seroma multi-omics to test reproducible modifiers of risk and progression. Candidate follow-up domains—including symptom-triggered imaging findings and exploratory systemic inflammatory markers—may be incorporated as prespecified research endpoints, but their predictive value and feasibility require rigorous evaluation before any consideration of routine use. Until such evidence is available, clinical management should remain aligned with established consensus statements and relevant regulatory communications. However, it is crucial to note that the theoretical stratification strategies discussed herein—such as baseline MRI screening and longitudinal follow-up with systemic inflammatory markers (e.g., CRP/ESR) for hypothesized high-risk subjects—are purely exploratory. These concepts are proposed solely to stimulate future prospective research and do not represent standardized or validated clinical recommendations. Routine clinical practice should continue to follow established, consensus-based guidelines from appropriate surgical and oncological societies for the management of BIA-ALCL. Viewed as a working G × E model, this framework changes practice primarily by reinforcing actions supported by current evidence: minimizing high-risk textured exposure when feasible, improving device documentation, and maintaining symptom-literate, guideline-concordant evaluation of late seromas or masses. In contrast, its most immediate impact is on research priorities: it motivates prospective cohorts linking verified device exposure histories with host immunogenetics, matched-normal tumor multi-omics, and stage-resolved capsule/seroma microenvironment profiling to test whether specific host factors or niche states reproducibly modulate risk and progression.

## 8. Conclusions: The Era of Genetic Biocompatibility

BIA-ALCL has moved from an unusual case-report signal to a defined clinicopathologic entity with actionable diagnostic and management pathways, and it now serves as a model for how long-lived biomaterials can reshape local immunobiology over time. Across populations, the most consistent exposure-level association remains with textured (particularly high-surface-area) implant surfaces; yet, the rarity of disease despite widespread exposure indicates that texture is best interpreted as a risk-amplifying niche rather than a deterministic cause. Mechanistic work supports a plausible inflammatory substrate—encompassing biofilm-associated microbial cues and sustained foreign-body responses—while also underscoring that such signals are neither unique to lymphoma nor sufficient to explain malignant progression in most individuals exposed to it.

Genomic studies have sharpened this multistep view by repeatedly identifying convergence on JAK/STAT pathway activation, consistent with strong selection for cytokine-responsive survival and proliferation programs within a chronically inflamed capsule environment. The frequent co-occurrence of alterations in epigenetic regulators further suggests that chromatin-state remodeling helps stabilize these signaling outputs and diversify phenotypes during progression from effusion-limited to more invasive disease. In parallel, evidence for immune-evasion circuitry—most notably frequent PD-L1 expression and recurrent *CD274* (9p24.1) copy number alterations—supports a model in which tumors not only arise in an inflammatory niche but can eventually convert that inflammation into checkpoint-mediated protection from immune clearance.

Clinically, this synthesis reinforces two practical realities: first, most patients present with localized disease and can be cured with complete surgical excision when recognized and staged appropriately, and second, prevention is currently best anchored in exposure mitigation and standardized, symptom-literate follow-up rather than speculative molecular screening. Regulatory actions—such as the FDA’s 2019 BIOCELL recall and subsequent strengthening of risk-communication requirements (including the boxed warning and patient decision checklist)—have operationalized this approach by prioritizing device-category risk reduction and informed consent as frontline preventive tools [79,89].

At the same time, emerging host-susceptibility signals motivate a research-grade “precision prevention” agenda: a cohort study suggests *BRCA1/2* carrier status may increase risk in textured-implant reconstruction settings, and an HLA allele association study supports the broader possibility that antigen presentation can tune susceptibility—both findings that now require multi-center, multi-ancestry replication with rigorous exposure documentation. The most decisive next steps include multi-center replication of candidate host signals (e.g., BRCA/HLA), matched-normal genomic studies to quantify true driver prevalence and exclude CHIP confounding, and integrated immune–stromal atlases across effusion-limited and invasive disease to connect genotype with microenvironmental selection and immune escape.

## Figures and Tables

**Table 1:** Summary of Key Epidemiologic Studies Stratifying BIA-ALCL Risk by Implant Surface Texture.

Study (Author, Year, Region) [Ref.]	Study Design & Population	Surface Classification Used	Key Findings (Risk Estimates/Observations)	Key Limitations Mentioned in Text
de Boer et al. (2018) [9] Netherlands	Population-based case–control study (nationwide pathology registry).	Macrotextured vs. microtextured vs. smooth	Quantified a strong, markedly increased relative risk for textured implants. Provided absolute risk estimates using population denominators.	Denominator estimation challenges (sales vs. implantation data).
Loch-Wilkinson et al. (2017) [11] Australia/New Zealand	Registry cohort analysis (integrated national registries).	High-surface-area (e.g., Biocell, polyurethane) vs. lower-surface-area textured	Reported significantly higher risks among recipients of higher-surface-area textured devices compared to lower-surface-area textured devices.	Incomplete exposure histories in some cases.
Cordeiro et al. (2020) [23] USA (Single Institution)	Prospective reconstruction cohort (long-term follow-up).	Macrotextured (Biocell) focused	Observed BIA-ALCL cases following prolonged exposure to macrotextured devices, yielding higher cumulative incidence estimates than sales-based approaches.	Single-institution data; findings may not generalize to all settings.
FDA MDR Analysis (2024) [13] USA (Regulatory Data)	Passive adverse event reporting system (MDR database).	Textured vs. smooth mentioned in reports	Explicitly notes that textured exposure dominates risk attribution among reported cases.	Subject to underreporting, duplicates, and missing denominators; cannot calculate true incidence.

**Table 2:** The Mutational Landscape of BIA-ALCL.

Gene Symbol	Mutation Type	Frequency (Approx.)	Pathogenic Mechanism (Role in BIA-ALCL)	Clinical Implication
*JAK1* [34]	Somatic (Gain-of-function)	15–40%	Constitutive Activation: Activates STAT3 without cytokine stimulation. Acts as the primary “engine” for cell proliferation.	Targetable by JAK inhibitors (e.g., Ruxolitinib).
*STAT3* [34,45]	Somatic (Gain-of-function)	30–60%	Nuclear Translocation: Directly upregulates cell survival genes (*BCL2*, *MYC*). Variants in SH2 domain (p.S614R, p.Y640F) are hotspots.	Diagnostic hallmark; high correlation with phosphorylated-STAT3 (pSTAT3) expression in IHC.
*TP53* [42,54]	Somatic/Germline	15–20%	Guardian Failure: Loss of DNA repair capability. Critical for the “Two-Hit” hypothesis. Allows cells with JAK/STAT errors to escape apoptosis.	Associated with poorer prognosis, genomic instability, and potential resistance to therapy.
*SOCS1/SOCS3* [54,55]	Somatic (Loss-of-function)/Hypermethylation	20–30%	Brake Failure: Loss of negative feedback loop that normally shuts down JAK/STAT signaling.	Explains why inflammation does not resolve naturally in these patients.
*DNMT3A* [20,54]	Somatic (Epigenetic)	10–15%	Epigenetic Drift: Associated with “Clonal Hematopoiesis (CHIP).” Suggests aging immune system prone to malignant transformation.	Links BIA-ALCL to age-related myeloid/lymphoid plasticity.
*KMT2C/D* [20,54]	Somatic (Loss-of-function)	20–25%	Chromatin Remodeling: alters gene accessibility, promoting stem-cell-like state in T-cells.	Contributes to persistence and survival of the malignant clone.

Note: Data compiled from major genomic profiling studies, specifically Oishi et al. (2018) [20], Blombery et al. (2018) [19] and Laurent et al. (2020) [35]. Percentages are approximate and represent aggregated reported cohorts.

**Table 3:** Potential Fluid-Phase Biomarkers for Distinguishing BIA-ALCL from Benign Late Seroma.

Biomarker Candidate	Biological Role in BIA-ALCL Niche	Finding in BIA-ALCL Effusions (vs. Benign) [Ref.]	Current Status
CD30 (Soluble form)	Shed from tumor cell surface; hallmark of activated lymphoid cells.	Significantly elevated. Key diagnostic marker (cytology/flow) [6,37].	Clinical Standard
IL-10	Immunoregulatory cytokine; promotes anergy and fosters a permissive niche.	Consistently elevated compared to reactive seromas [56,58].	Research/Emerging
IL-13	Type 2 cytokine linked to allergic response, fibrosis, and STAT6 activation in tumor cells.	Elevated; linked to tumor-immune co-evolution and tissue remodeling [31,56].	Research/Emerging
Eotaxin (CCL11)	Eosinophil chemoattractant; consistent with allergic/Th2-skewed inflammation.	Elevated; correlates with eosinophilic infiltration often seen in histology [56].	Research
IL-6	Pro-inflammatory cytokine driving STAT3 activation.	Variable; IL-10/IL-6 ratio proposed as discriminative [56].	Research

**Table 4:** Exploratory framework for future validation studies: host-context strata, implant-surface exposure considerations, and candidate follow-up domains.

Conceptual Stratum (Research Only)	Illustrative Host-Context Features	Implant-Surface Exposure Considerations to Examine in Future Studies	Candidate Follow-Up Domains/Outcomes for Prospective Validation
Lower-susceptibility hypothesis	No reported family history of lymphoma; no autoimmune history; no known pathogenic variants in candidate pathways	Compare outcomes across ISO-defined surface categories and quantify implant-surface exposure history where available. Treat prior regulatory safety communications as contextual variables for analysis rather than directives.	Symptom-driven clinical events (e.g., late seroma, capsular findings) and standardized adverse-event reporting. Imaging findings may be collected in research settings as prespecified endpoints.
Intermediate-susceptibility hypothesis	Family history suggestive of immune dysregulation or autoimmunity; variant of uncertain significance in candidate genes	Test effect modification by surface category in stratified cohorts. Where applicable, analyze macrotextured exposure as an observational exposure group in patients already implanted with such devices.	Longitudinal clinical and imaging endpoints may be explored; modality and interval should be defined a priori for validation studies.
Higher-susceptibility hypothesis	Confirmed pathogenic germline variants in cancer predisposition genes; prior lymphoma history	Prioritize complete documentation of reconstruction modality and surface exposure to enable sensitivity analyses. Autologous reconstruction can be included as a comparator subgroup when clinically selected.	Exploratory biomarker and immunologic endpoints (including inflammatory markers) and imaging can be evaluated as candidate research outcomes; clinical utility requires prospective validation.

This table is intended solely as a hypothesis-generating framework to inform prospective study design. It should not be interpreted as clinical guidance, a device-selection recommendation, or a surveillance protocol. The listed host-context features are illustrative and are not validated predictors of BIA-ALCL risk. No follow-up schedule is implied. Clinical management should follow established consensus statements and relevant regulatory communications. ISO, International Organization for Standardization; BIA-ALCL, breast implant-associated anaplastic large cell lymphoma.

**Table 5:** Timeline and Summary of Key Regulatory Actions Regarding BIA-ALCL.

Regulatory Body/Region	Key Actions & Dates	Current Status for Macrotextured/High-Surface-Area Devices	Key Recommendations for Patients/Clinicians
FDA (USA)	2019: Requested a voluntary recall of specific macrotextured devices (Allergan BIOCELL) following safety signals indicating disproportionate BIA-ALCL risk [13,79]. 2021: Updated labeling requirements for all breast implants, including a boxed warning and the use of a patient decision checklist to support informed consent [14,78]. (An example checklist format is available from ASPS [87].)	Specific recalled devices withdrawn. Other textured implants remain available, accompanied by stringent warning labels.	FDA communications state that prophylactic removal is not recommended for asymptomatic patients [90]. Emphasis is placed on risk disclosure, symptom awareness, and clinician–patient discussion, with evaluation guided by clinical presentation [78].
ANSM (France)	2019: Issued a decision banning the placing on the market, distribution, and use of macrotextured and polyurethane-coated breast implants [80].	Banned from use in reconstruction and augmentation.	Smooth implants are the default. Prophylactic explantation is not recommended for women without symptoms.
TGA (Australia)	2019: Following review, took regulatory action to cancel or suspend specific high-surface-area textured implants and expanders from the Australian Register of Therapeutic Goods [81].	Highly restricted; specific high-risk textured devices removed from the market.	Underscores that implant surface risk is consistent across jurisdictions. Emphasizes risk communication and vigilance for symptoms; prophylactic removal is not recommended for asymptomatic individuals [91].
Health Canada	2019: Suspended the medical device licenses for certain macrotextured breast implants (Allergan BIOCELL) following a safety review identifying higher risk [82].	Specific licenses for targeted macrotextured devices suspended.	Advisories emphasize symptom vigilance and indicate that preventive removal is not recommended in the absence of signs or symptoms suggestive of BIA-ALCL [82].
EMA/EU Scientific Committees	Ongoing: EU scientific bodies (e.g., SCHEER) acknowledge the risk association with texture but indicate textured surfaces may still be clinically necessary for certain reconstructive indications.	Varies by individual member state (e.g., France’s ban vs. other nations’ heightened surveillance).	Approaches vary by member state; overarching themes include multidisciplinary evaluation and strengthened informed consent.

## Data Availability

No new data were created or analyzed in this study. Data sharing is not applicable to this article.

## Abbreviations

The following abbreviations are used in this manuscript:

ALK	Anaplastic Lymphoma Kinase
ANSM	Agence Nationale de Sécurité du Médicament et des produits de santé (French National Agency for the Safety of Medicines and Health Products)
BIA-ALCL	Breast Implant-Associated Anaplastic Large Cell Lymphoma
BRCA	Breast Cancer gene
CD	Cluster of Differentiation (e.g., CD30, CD274)
CHIP	Clonal Hematopoiesis of Indeterminate Potential
CNA	Copy-Number Alteration
CRP	C-Reactive Protein
EMA	European Medicines Agency
ESR	Erythrocyte Sedimentation Rate
FDA	U.S. Food and Drug Administration
G × E	Gene–Environment
HLA	Human Leukocyte Antigen
IL	Interleukin
ISO	International Organization for Standardization
JAK	Janus Kinase
MDR	Medical Device Reports
MDT	Multidisciplinary Team
MRI	Magnetic Resonance Imaging
NBIR	National Breast Implant Registry
PD-1	Programmed Cell Death Protein 1
PD-L1	Programmed Death-Ligand 1
PET/CT	Positron Emission Tomography/Computed Tomography
PROFILE	Patient Registry and Outcomes for Breast Implants and Anaplastic Large Cell Lymphoma Etiology and Epidemiology
ROS	Reactive Oxygen Species
SCHEER	Scientific Committee on Health, Environmental and Emerging Risks
STAT	Signal Transducer and Activator of Transcription
TCR	T-Cell Receptor
TGA	Therapeutic Goods Administration (Australia)
Th	T helper (cell)
TP53	Tumor Protein p53
US	Ultrasound
VUS	Variant of Uncertain Significance
WES	Whole-Exome Sequencing
WHO	World Health Organization
YAP	Yes-Associated Protein
